# Sublethal Doxorubicin Promotes Extracellular Vesicle Biogenesis in A375 Melanoma Cells: Implications for Vesicle-Loaded TGF-β-Mediated Cancer Progression and Cardiovascular Pathophysiology

**DOI:** 10.3390/ijms26178524

**Published:** 2025-09-02

**Authors:** Laura Fernanda Fernández-Fonseca, Susana Novoa-Herrán, Adriana Umaña-Pérez, Luis Alberto Gómez-Grosso

**Affiliations:** 1 Master in Biochemistry Program, Department of Chemistry, Science Faculty, Universidad Nacional deColombia, Bogotá 111321, Colombia; 2 Molecular Physiology Group, Sub-Direction of Scientific and Technological Research, Direction of Public Health Research, National Institute of Health, Bogotá 111321, Colombia; snovoa@ins.gov.co; 3 Department of Chemistry, Science Faculty, Universidad Nacional de Colombia, Bogotá 111321, Colombia; yaumanap@unal.edu.co; 4 Department of Physiological Sciences, Faculty of Medicine, Universidad Nacional de Colombia, Bogotá 111321, Colombia

**Keywords:** extracellular vesicles, doxorubicin, melanoma, TGF-β, atherogenesis, cardiac fibrosis, metabolic remodeling, chemotherapy-induced toxicity, tumor microenvironment, cytokines, cardiovascular diseases

## Abstract

Doxorubicin (Dox) is not a first-line treatment for melanoma due to limited antitumor efficacy and dose-dependent cardiotoxicity. However, sublethal doses may trigger adaptive cellular responses that influence tumor progression and systemic toxicity. Small extracellular vesicles (EVs) are key mediators of intercellular communication and can carry bioactive molecules that modulate both the tumor microenvironment and distant tissues. This study investigates how sublethal Dox exposure alters EV biogenesis and cargo in A375 melanoma cells and explores the potential implications for cardiovascular function. We treated human A375 melanoma cells with 10 nM dox for 96 h. EVs were isolated using differential ultracentrifugation and size exclusion chromatography. Vesicle characterization included Immunocytochemistry for CD63, CD81, CD9, Rab7 and TSG101, scanning electron microscopy (SEM) Nanoparticle Tracking Analysis (NTA), and Western blotting for CD81 and CytC. We analyzed cytokine content using cytokine membrane arrays. Guinea pig cardiomyocytes were exposed to the isolated vesicles, and mitochondrial activity was evaluated using the MTT assay. Statistical analysis included *t*-tests, ANOVA, Cohen’s *d*, and R^2^ and η^2^. Dox exposure significantly increased EV production (13.6-fold; *p* = 0.000014) and shifted vesicle size distribution. CD81 expression was significantly upregulated (*p* = 0.0083), and SEM (microscopy) confirmed enhanced vesiculation. EVs from treated cells were enriched in TGF-β (*p* = 0.0134), VEGF, CXCL1, CXCL12, CCL5, IL-3, IL-4, IL-10, Galectin-3, and KITLG. Cardiomyocytes exposed to these vesicles showed a 2.3-fold reduction in mitochondrial activity (*p* = 0.0021), an effect absent when vesicles were removed. Bioinformatic analysis linked EV cargo to pathways involved in cardiac hypertrophy, inflammation, and fibrosis. As conclusion, sublethal Doxorubicin reprograms melanoma-derived EVs by enhancing their production and enriching their cargo with profibrotic and immunomodulatory mediators. These vesicles may contribute to tumor progression and cardiovascular physiopathology, suggesting that targeting EVs could improve therapeutic outcomes in cancer and cardiovascular disease.

## 1. Introduction

Researchers have historically used Doxorubicin (Dox) to treat melanoma; however, they no longer consider it a first-line chemotherapeutic agent because of its limited antitumor efficacy and well-documented dose-dependent cardiotoxicity [1,2,3]. The emergence of more effective therapies, such as immune checkpoint inhibitors (e.g., pembrolizumab, nivolumab) and targeted treatments (e.g., B-Raf inhibitors), has shifted the clinical approach to melanoma, offering improved survival, higher response rates, and fewer adverse effects, especially in advanced stages [4]. Nevertheless, Dox may still be employed in selected cases where other options fail, though resistance remains a significant obstacle to its effectiveness [1,5]. Melanoma cells exhibit intrinsic resistance mechanisms that reduce Dox’s therapeutic impact [6], while its toxicity, particularly, its harmful effects on the heart, further limits its clinical utility [6,7].

Small extracellular vesicles (EVs) have recently gained attention as key mediators of intercellular communication [8]. These vesicles transport bioactive molecules such as RNA, cytokines, and growth factors, influencing tumor progression, immune modulation, and resistance to therapy [9,10]. Increasing evidence suggests that EVs participate in long-range communication between tumor cells, supporting processes such as cellular reprogramming and remodeling of the tumor microenvironment [11]. Chemotherapeutic stress, including Dox exposure, has been shown to enhance the secretion of EVs, particularly exosomes, which carry molecular cargo capable of altering both local and systemic cellular responses [12,13].

Understanding the biogenesis of EVs is essential for uncovering their roles in cell-to-cell communication and their potential as therapeutic targets in cancer and beyond. However, the mechanisms governing their formation and secretion remain only partially understood [14,15]. The overlap in molecular markers and biophysical properties among different EV populations complicates their classification, as many shares common proteins such as CD9, CD63, and CD81, regardless of their origin. Whether EVs arise through endosomal pathways, forming intraluminal vesicles that become exosomes, or via direct budding from the plasma membrane to produce ectosomes is still under investigation. Tracking their intracellular routes using specific markers has provided insights into their biogenesis and cargo sorting from the endoplasmic reticulum through late endosomes or the plasma membrane [16]. Additionally, there is evidence that cargo selection may occur during EV formation and that some exported molecules may actively participate in this process [17].

Although their biological relevance is increasingly recognized, the mechanisms regulating the release of EVs and their molecular composition, particularly under chemotherapeutic pressure, remain largely unelucidated [18]. One of the main challenges in EV research is their heterogeneity, as subpopulations often share surface markers and functional characteristics, which hinders their precise identification [19]. Moreover, EVs released by cancer cells, including those with melanoma, may influence distant non-malignant cells, such as cardiomyocytes, potentially contributing to the development of chemotherapy-induced cardiotoxicity [7,20,21].

Interestingly, some studies associate exosomes with cardioprotective effects, especially under hypoxic conditions or ischemic stress [22,23,24,25]. However, vesicles released under pathological or stress-induced conditions may have detrimental cardiac effects [5]. For instance, we have implicated EVs derived from breast tumor cells and those released upon Dox exposure as mediators of Dox-induced cardiotoxicity [7]. Nevertheless, the specific effects of Dox on EV biogenesis and function in melanoma remain largely unexplored.

This study investigates the effects of sublethal Dox exposure on the biogenesis and molecular composition of EVs in human melanoma A375 cells, aiming to elucidate their role in mediating both local and systemic effects of chemotherapy. While researchers have well established that Dox induces cellular stress responses that contribute to tumor evolution and resistance, they know less about how cells communicate these responses via EVs and how they affect nontumor tissues [26]. We hypothesize that sublethal Dox treatment enhances the release of vesicles enriched in specific molecular cargo, particularly cytokines, that influence both melanoma progression and cardiovascular health.

Cytokines packaged within EVs play pivotal roles in modulating the tumor microenvironment. For example, transforming growth factor beta (TGF-β), a multifunctional cytokine frequently associated with epithelial–mesenchymal transition (EMT), immune suppression, and extracellular matrix remodeling, has been implicated in promoting cancer invasiveness and metastatic potential [24,27]. In the context of EV-mediated signaling, TGF-β can exert its effects not only within the tumor niche but also at distant sites by being selectively enriched in vesicles and delivered to target cells [7]. In this study, we specifically assess the Dox-induced enrichment of TGF-β and other pro-tumorigenic cytokines, including vascular endothelial growth factor (VEGF), Stromal cell-derived factor 1 (CXCL12), Growth-regulated alpha protein (CXCL1), C-C motif chemokine 5 (CCL5), Interleukin-3 (IL-3), Interleukin-4 (IL-4), Interleukin-10 (IL-10), Galectin-3, and Kit ligand (KITLG), which may facilitate angiogenesis, immune evasion, and stromal activation—hallmarks of tumor progression.

Beyond their oncogenic roles, these EV-associated cytokines may also impact cardiovascular physiology. TGF-β is a well-established driver of non-ischemic cardiac fibrosis, stimulating the activation of fibroblasts and collagen deposition, which contributes to ventricular stiffening and the progression of heart failure [28,29,30]. Moreover, researchers have linked TGF-β and Galectin-3 to atherosclerosis due to their roles in endothelial dysfunction, smooth muscle proliferation, and chronic vascular inflammation [29,31]. Similarly, proangiogenic factors, such as VEGF, while crucial for tumor vascularization, may disrupt vascular homeostasis when systemically distributed, thereby increasing vascular permeability and contributing to vascular remodeling. Furthermore, IL-4 and IL-10, typically classified as anti-inflammatory cytokines, have context-dependent effects that may either protect or exacerbate tissue remodeling and metabolic dysfunction in cardiomyocytes [32,33,34].

To investigate these hypotheses, we conducted a comprehensive characterization of EVs released by melanoma A375 cells following sublethal Dox treatment, using nanoparticle tracking analysis, electron microscopy, and immunoblotting for canonical EV markers (CD9, CD63, CD81, Tumor susceptibility gene 101 protein TSG101). We quantified cytokine content using cytokine arrays, with a focus on those with established roles in cancer and cardiovascular biology. Importantly, we assessed the functional impact of these vesicles on primary cardiomyocytes, measuring alterations in cellular viability and metabolic activity.

By linking changes in vesicle cargo composition to downstream effects on cardiovascular physiology, this study aims to uncover novel mechanisms through which melanoma-derived EVs may contribute to chemotherapy-induced cardiotoxicity. Our findings could provide a mechanistic link between cancer progression [35] and cardiovascular disease, particularly highlighting how TGF-β-enriched vesicles may simultaneously promote melanoma aggressiveness and induce metabolic cardiomyocyte changes, as well as adverse atherogenic stimuli and non-ischemic cardiac fibrosis in the cardiovascular system. These insights could inform the development of targeted interventions to modulate vesicle biogenesis or cargo loading, thereby limiting off-target toxicity while preserving chemotherapy’s antitumor efficacy.

## 2. Results

### 2.1. Doxorubicin Treatment Alters A375 Human Melanoma Cell Viability Without Inducing Apoptosis/Necrosis: Establishing an Optimal Sublethal Concentration for EV Studies

**Figure 1:**
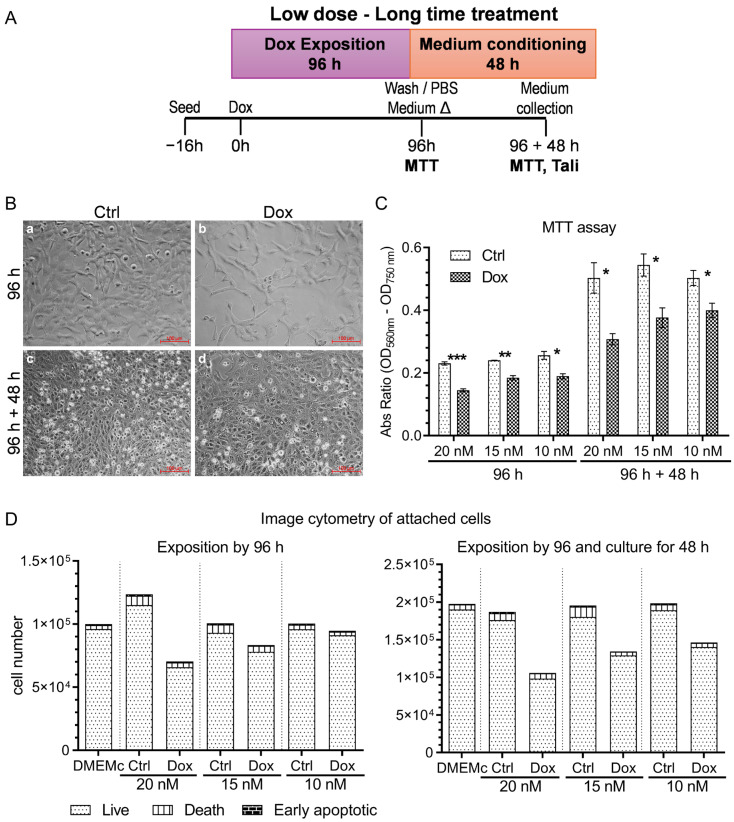
**Overview of the effects of Doxorubicin treatment on A375 human melanoma cells.** (**A**) Experimental timeline presenting Doxorubicin (Dox) exposition and conditioned media collection strategy. Schematic representation of the experimental workflow used to treat A375 cells with Dox and collect conditioned media for downstream analyses. We exposed cells to Dox (10–100 nM) in complete medium for 96 h, followed by a 48 h incubation in Dox- and serum-free medium (medium Δ: medium change). We evaluated cellular responses at two key time points: after 96 h of treatment and after an additional 48 h culture period, for each Dox concentration and its respective vehicle control (DMSO). (**B**) Morphological analysis of A375 cells following Dox treatment. Representative phase contrast micrographs showing cell morphology at 96 h (panels **a** and **b**) and after the 48 h post-treatment period (panels **c** and **d**). We acquired images using a Nikon Eclipse Ti-S inverted microscope equipped with a Digital Sight DS-Fil camera and a 20× Ph1 phase contrast objective. Panel show (**a**) vehicle control at 96 h of Dox exposition; (**b**) 10 nM Dox at 96 h; (**c**) control after an additional 48 h in Δ medium; (**d**) Dox-treated cells after an additional 48 h. Representative of five fields per condition; scale bar 100 µm. (**C**) Impact of Dox on A375 cell viability and proliferation. MTT assay results demonstrating dose- and time-dependent reductions in metabolic activity following Dox treatment. We corrected and normalized absorbance values to vehicle controls. We present data as mean ± SEM (standard error of the mean) from three technical replicates, representative of three independent biological experiments (n = 3). We performed statistical comparisons between each Dox concentration and its corresponding control using unpaired two-tailed Student’s *t*-tests. *p* < 0.05 (*), *p* < 0.01 (**), *p* < 0.001 (***). (**D**) Assessment of cell death by apoptosis and necrosis. Quantification of apoptosis and necrosis using Annexin V and propidium iodide staining followed by image cytometry. We analyzed cells after 96 h of Dox treatment (Δ medium) and after an additional 48 h of culture (collection medium). We collected fluorescence data from 20 randomly selected fields, counting between 30,000 and 80,000 cells per condition. We present the results as mean percentages from two technical replicates, representative of three independent biological experiments (n = 3). We used unstained cells as negative controls.

We first designed and implemented an experimental workflow to identify a sublethal concentration of Doxorubicin (Dox) that could induce cellular stress without triggering apoptosis or necrosis, enabling us to collect conditioned media for analyzing small extracellular vesicle (EVs) biogenesis (Figure 1A). We exposed A375 melanoma cells to varying concentrations of Dox in complete medium (DMEM with 10% fetal bovine serum—FBS) for 96 h, then washed them twice with Phosphate Buffered Saline (PBS) and incubated them for an additional 48 h in serum-depleted medium, to avoid contamination of subsequent collected A375 conditioned medium with bovine EVs and Dox residues. We incubated A375 cells without FBS to prevent contamination with bovine-derived EVs and soluble factors. To further ensure removal of residual serum components, we washed the cell cultures twice with PBS before conditioning.

We assessed the cell viability using MTT assays, image cytometry with apoptosis/necrosis staining, and light microscopy. We determined the effect of Dox treatment on growth and cell death rates after 96 h of exposure and an additional 48 h of culture (Figure 1A).

The initial MTT and image cytometry assays revealed a dose-dependent reduction in viability across the Dox concentration range (100–12.5 nM). Higher Dox concentrations (e.g., 100 nM) significantly reduced cell viability (96 h: F.C. = −4.19, *p* < 0.0001, Cohen’s *d* = 13.98; 96 h + 48 h: F.C. = −5.41, *p* = 0.0022, Cohen’s *d* = 4.87), whereas 12.5 nM moderately reduced proliferation without causing significant morphological changes or cell death (96 h: F.C. = −1.50, *p* = 0.0068, Cohen’s *d* = 4.19; 96 h + 48 h: F.C. = −1.47, *p* = 0.0281, Cohen’s *d* = 2.75) (Supplementary Figure S1C,D), corroborated by light microscopy (Supplementary Figure S1A,B). We then assayed Dox at 20, 15, and 10 nM, obtaining similar results to the 12.5 nM dose. Cell count results from image cytometry (Figure 1D), together with the MTT assay results (Figure 1C), indicated a dose-dependent reduction in cell metabolism and number in Dox-treated cultures compared to control cultures, without morphological alterations (Figure 1B). We observed a significant decrease in metabolic activity (MTT reductase activity) after Dox exposure by 96 h (*p*-value: 20 nM: 0.0003, Cohen’s *d* = 9.75; 15 nM: 0.0014, Cohen’s *d* = 6.43; 10 nM: 0.0109, Cohen’s *d* = 3.66), with a further reduction after an additional 48 h of culture (*p*-value: 96 h treatment 20 nM: 0.0196, Cohen’s *d* = 3.08; 15 nM: 0.0234, Cohen’s *d* = 2.91; 10 nM: 0.0355, Cohen’s *d* = 2.55). The image cytometry of Annexin V and propidium iodide (PI) staining showed there were no significant differences between the level of apoptosis/necrosis of cells treated with Dox and their respective control (Figure 1D). These results showed that 10 nM Dox induced a controlled, nonlethal stress condition in A375 melanoma cells, ideal for downstream analyses of exosomes and other small EVs biogenesis and cargo.

### 2.2. Doxorubicin at 10 nM Modulates Intracellular Vesicle Markers and Promotes Endosomal Activity in A375 Cells

**Figure 2:**
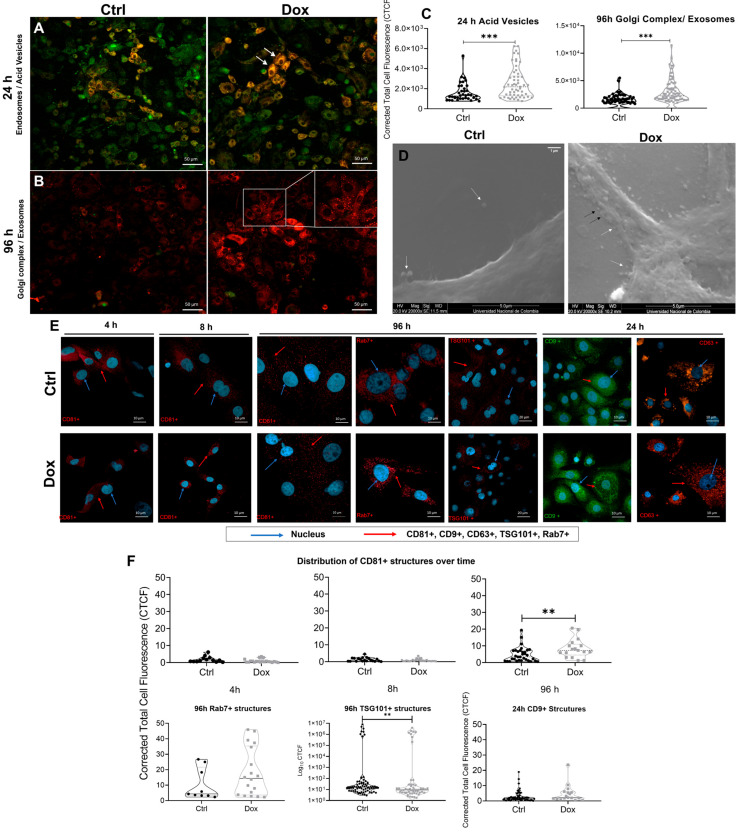
**Doxorubicin (10 nM) alters intracellular vesicle marker distribution and enhances extracellular vesicle biogenesis in A375 melanoma cells**. (**A**) Live-cell visualization of acidic vesicles 24 h post-Dox treatment (n = 2). Acridine orange staining revealed an increased number of acidic vesicles in Dox-treated cells (white arrows), consistent with enhanced endolysosomal activity; scale bar 50 µm. (**B**) Golgi complex remodeling and ceramide-rich vesicles after Dox exposure. Bodipy-TR ceramide staining at 96 h showed redistribution and clustering of Golgi-associated vesicles in Dox-treated cells. Highlighted regions indicate ceramide-enriched structures, potentially representing exosome precursors. We acquired images during live-cell imaging sessions (n = 2), representative of three fields per condition: scale bar 50 µm. (**C**) Quantification of vesicle marker fluorescence. Corrected total cell fluorescence (CTCF) was calculated for acridine orange and Bodipy-TR ceramide signals, using the formula: CTCF = Integrated Density − (Area × Background Mean Fluorescence). Analysis included 40–50 cells per group, revealing a significant increase in acidic and ceramide-positive vesicles in cells treated with Dox; scale bar 10 µm. (**D**) SEM imaging (scanning electron microscopy: images (microscopy) of extracellular vesicles Ctrl and Dox (n = 1). Scanning electron microscopy revealed heterogeneous populations of vesicles on the A375 cell surface 24 h after Dox and without treatment. Larger vesicles (white arrows) and smaller vesicles (black arrows) were identified, with diameters ranging from 131 to 158 nm consistent with EVs/exosome dimensions: scale bar 5 µm. (**E**) Temporal analysis of EVs-related protein markers CD81, CD9, CD63, TSG101, and Rab7. Immunofluorescence assays at 4, 8, 24, and 96 h post-treatment showed an increased expression of CD81 (exosomes marker), Rab7 (late endosome marker), and TSG101 (in Dox-treated cells, particularly at 96 h); (**F**) Statistical analysis of CD81, Rab7, TSG101 (marker expression; the y-axis is presented in logarithmic scale (log_10_) to represent better the intensity of TSG101 which presents a high value for some cells in a biological assay, *p* = 0.0068). CTCF values for each time point were analyzed using two-tailed unpaired Mann–Whitney tests (n = 6). We observed significant increases in CD81 (*p* = 0.0083) Dox-treated cells, denoted as *p* < 0.01 (**), *p* < 0.001 (***).

To assess whether 10 nM Dox influences vesicle biogenesis pathways, we evaluated late endosomes (LEs), multi-vesicular bodies (MVBs), and Golgi-associated vesicles using acridine orange, Bodipy-TR ceramide, and immunostaining for CD81 and Rab7 (Figure 2).

Treatment of A375 melanoma cells with 10 nM Dox induced marked alterations in intracellular vesicle organization and biogenesis. Acridine orange staining at 24 h revealed a notable increase in acidic vesicular compartments in treated cells compared to controls (Figure 2A). Quantitative image analysis showed that the corrected total cell fluorescence (CTCF) of acridine orange significantly increased in Dox-treated cells (Corrected total cell fluorescence CTCF = 2.12 × 10^3^ ± 1.54 × 10^3^) relative to untreated controls (CTCF = 1.23 × 10^3^ ± 9.13 × 10^2^), with a fold change (F.C.) of 1.72 and a *p*-value of 0.0007, R^2^ (η^2^) = 0.132; n ≈ 70 cells, suggesting enhanced acidification and maturation of endolysosomal vesicles.

Bodipy-TR ceramide staining at 96 h confirmed increased Golgi-associated vesicle accumulation (Figure 2B). The Dox-treated cells showed higher CTCF values (CTCF = 2.24 × 10^3^ ± 2.26 × 10^3^) versus controls (CTCF = 1.30 × 10^3^ ± 1.14 × 10^3^), with a fold change of 1.72 and a *p*-value of <0.0001, R^2^ (η^2^) = 0.203; n ≈ 70 cells (Figure 2C). These observations indicated an upregulation of vesicle trafficking from the Golgi, likely contributing to exosome precursor generation. Live imaging captured the dynamic clustering of ceramide-rich membranes, consistent with ceramide-mediated exosome biogenesis mechanisms.

Scanning electron microscopy (SEM) at 24 h post-treatment revealed a surface enrichment of extracellular vesicles in Dox-exposed cells. The vesicle population was morphologically heterogeneous, with large vesicles (highlighted by white arrows) and small vesicles (black arrows) measuring between 131 and 158 nm, consistent with the size range of small EVs such as exosomes (Figure 2D). This structural data provided visual confirmation of enhanced vesicle release under sublethal chemotherapeutic stress.

Immunofluorescence analysis of CD81, CD9, CD63, TSG101, and Rab7 vesicle markers provided additional evidence of EVs biogenesis. In Dox-treated cells, CD81 and TSG101 expression increased significantly at 96 h (F.C. = 2.13, *p* = 0.0083 and F.C = 0.82, *p*= 0.0068, respectively), while Rab7 levels also rose markedly (F.C. = 3.34, *p* = 0.348), indicating stimulation of both exosome-associated and late endosomal pathways. The time-course analysis confirmed that the induction of Rab7 occurred earlier (4–8 h), whereas, CD81 peaked later, consistent with sequential vesicle maturation and trafficking events (Figure 2E,F).

The results suggested that 10 nM Dox promoted vesicular remodeling in A375 cells by enhancing acidic vesicle formation, stimulating Golgi-derived trafficking, and increasing expression of EVs-related markers. These quantitative and structural data supported the notion that Dox triggered a robust increase in endosomal activity and exosome small EVs biogenesis under sublethal stress conditions.

### 2.3. Isolation and Characterization of EVs from Doxorubicin-Treated A375 Cells

**Figure 3:**
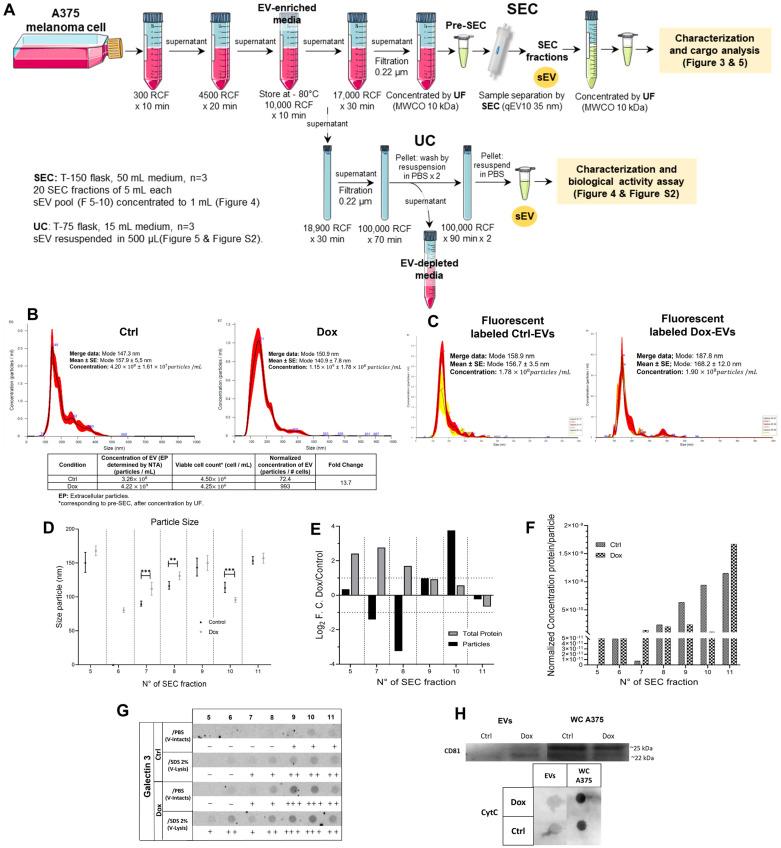
**Isolation, quantification, and characterization of small extracellular vesicles derived from A375 melanoma cells treated with Doxorubicin**. (**A**) Workflow for EVs isolation. Schematic representation of the EVs isolation protocol from A375 conditioned media collected after 48 h of treatment with 10 nM Doxorubicin. The protocol combined differential ultracentrifugation (UF) with size exclusion chromatography (SEC) to enrich EVs fractions. (**B**) We used nanoparticle tracking analysis (NTA) to quantify EVs from pre-SEC fractions (18,000× *g*) and post-SEC fractions derived from control and Dox-treated A375 cells. The histogram shows the mean particle concentration (black line), the standard error of the mean (red), and statistical significance values (white font) from five independent measurements (n = 2). The accompanying table reports particle concentrations normalized to the number of viable donor cells (per 10^6^ cells, determined by trypan blue exclusion) and to total protein content (per µg), as well as the fold change (F.C.) between Dox and control conditions. (**C**) Fluorescence-NTA of pooled EVs fractions (5–10). Bodipy-TR- labeled EVs showed a distinct fluorescent signal in the Dox-treated samples, consistent with ceramide-enriched EVs. The graph displays mean signal (yellow line), SEM (red) (statistical), and relative fluorescence (orange), with statistical annotations. (**D**) Particle size distribution across SEC fractions 5–11. NTA revealed consistent EVs size (80–168 nm) across fractions, with significantly higher particle counts in Dox-treated samples (F7: *p* = 0.00001, F8: *p* = 0.0047, F10: *p* = 0.00001), denoted as *p <* 0.01 (**), *p <* 0.001 (***). (**E**) Log_2_ fold change in protein and particle concentration. Comparison of Dox versus control in SEC fractions 5–11 revealed enrichment in both protein and particle content in the Dox group. (**F**) Fold change in particle concentration × mL^−1^. Dox-treated cells released a higher concentration of EVs across key SEC fractions, indicating enhanced vesicle secretion. (**G**) Protein-to-particle ratio. Normalized protein content per vesicle showed differences between Dox and control groups, suggesting altered cargo loading under Dox treatment “−” symbol denote absence of signal, while signal intensity is shown by the number of “+”. (**H**) Dot blot for Galectin-3 in EVs fractions 5–11. Detection of Galectin-3, a marker of intact and lysed vesicles, confirmed its presence in both conditions, with stronger signals in Dox-treated fractions. (**I**) Western blot and dot blot analysis of pooled EVs (fractions 5–10). CD81 expression was elevated in Dox-derived EVs and absence of Cytochrome C (CytC), supporting increased exosome marker content in treated cells and minimal contamination. Relative centrifugal forces (RCF) = g-force; extracellular particles (EP).

We isolated EVs from conditioned media of control and Dox-treated A375 cells using differential centrifugation followed by size exclusion chromatography (SEC). Figure 3A summarizes the isolation workflow and characterization of small EVs derived from A375 melanoma cells exposed to 10 nM Dox. The protocol combined differential ultracentrifugation (100,000× *g*) and SEC to enrich vesicle fractions from conditioned media collected after 48 h of treatment. This approach allowed for a refined separation and quantitative evaluation of the vesicle populations.

Quantification by nanoparticle tracking analysis (NTA) (Figure 3B) revealed that Dox-treated cells secreted significantly more vesicles than controls. Dox-treated cultures released in two independent biological experiments (n = 2) mean 3.26 × 10^9^ ± 4.47 × 10^7^ particles/mL, compared to 2.40 × 10^8^ ± 1.21 × 10^7^ particles/mL in the untreated group (13.6-fold; *p* = 0.000014; Cohen’s *d* = 106.3; R^2^ (η^2^) = 0.052, significant effect). The table included in Figure 3B presents EVs yield normalized to the number of viable cells, which supports the reported ~13.6-fold increase in vesicle secretion by Dox-treated cells. This confirms that the elevated vesicles secretion is not solely attributable to the changes in cell viability. We used fluorescence-NTA with Bodipy-TR labeling to confirm that these vesicles were enriched in ceramide-associated membranes, indicating a Golgi or endosomal origin. The fluorescent signal increased 1.07-fold in Dox-treated samples, with a large effect size (Cohen’s *d* = 1.2; R^2^ (η^2^) = 0.736), supporting the notion of stress-induced changes in vesicle biogenesis pathways (Figure 3C).

The size distribution of particles across SEC fractions 5–11 remained within the expected exosomal range (80–168 nm). Still, counts were significantly higher in the Dox group in particle size within fractions 7, 8, and 10, compared to control EVs in the same fractions (F7: *p* = 0.00001, F8: *p* = 0.0047, F10: *p* = 0.00001). These fractions correspond to the elution range, typically enriched in small EVs. Fractions 5, 6, and 11 showed no statistically significant differences between conditions. ANOVA confirmed a robust treatment effect (η^2^ = 0.145, significant effect), with multiple post hoc comparisons reaching significance *(p* < 0.05 to *p* < 0.001). Further analysis of log_2_ fold changes in protein and particle content in fractions 5–10 (Figure 3E) revealed consistent enrichment in the Dox group in total protein, particularly in fractions 9 and 10, where the particle fold change reached a peak of 3.76. These results were supported by large effect sizes (Cohen’s *d* = 1.06), reflecting reproducible differences across replicates.

The increase in EVs protein content per particle across SEC fractions was also evident, with a significant 19-fold increase observed in fraction 7 of Dox-treated cells (Figure 3F), corroborated by R^2^ (η^2^) = 0.006, indicating a significant effect. Normalizing protein content to particle number revealed shifts in cargo loading in the Dox group, suggesting qualitative changes in EVs composition (Figure 3G).

When we analyzed the dot blot for Galectin-3 in fractions 5–11, we confirmed protein enrichment in these fractions. Notably, fraction 9 showed increased Galectin-3 signal with a Cohen’s *d* of 0.91. Western blot analysis of pooled fractions (5–10) further revealed elevated expression of CD81, a canonical exosome marker, in vesicles from Dox-treated cells, with an estimated fold increase of ~2.1 and Cohen’s *d* = 0.84. Finally, a dot blot for Cytochrome C (CytC), a representative negative marker, included EV-enriched pooled fractions and whole-cell lysates (WC) from A375 control cells. The absence of CytC in the EV fractions supports minimal contamination from intracellular components.

Together, these data suggest that 10 nM Dox promoted the secretion of vesicles with altered biophysical and biochemical features, including higher protein content, increased marker expression, and ceramide enrichment. These changes are consistent with enhanced vesicle biogenesis and stress-induced remodeling of EVs cargo, potentially influencing their downstream biological functions in the tumor microenvironment or distant tissues.

### 2.4. Doxorubicin-Induced EVs from A375 Cells Alter Metabolism in Primary Cardiomyocytes

To investigate the functional impact of melanoma-derived EVs on cardiac metabolism, we exposed primary ventricular cardiomyocytes from guinea pigs to small EVs isolated from A375 melanoma cells treated with 10 nM Dox (Dox-EVs) or control (Ctrl-EVs). Using single-cell MTT assays, we evaluated the effects on mitochondrial reductase activity as a proxy for metabolic function.

**Figure 4:**
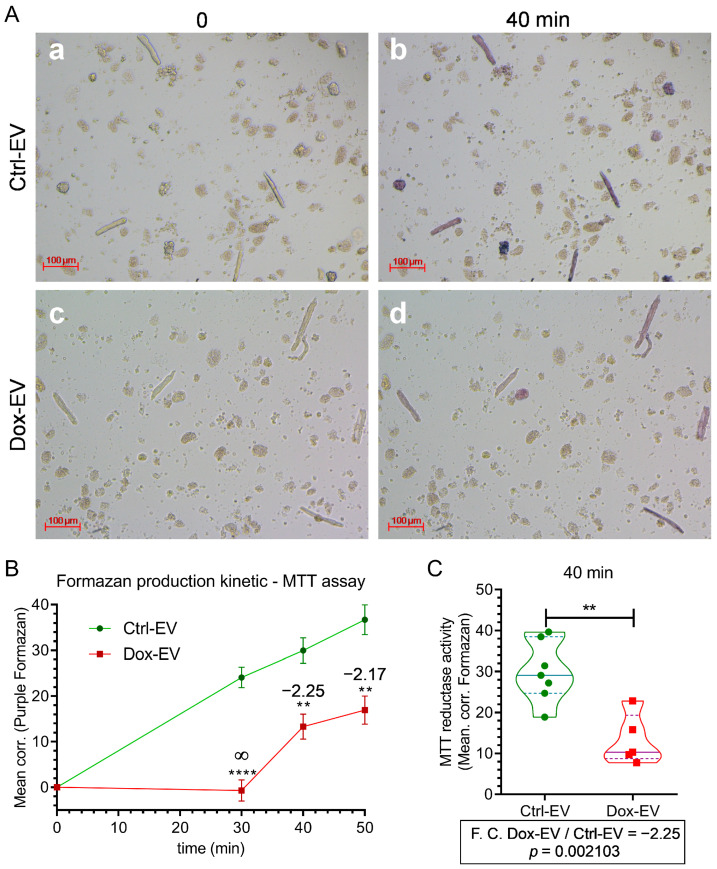
**Doxorubicin-induced EVs impair mitochondrial metabolism in primary cardiomyocytes**. (**A**) Bright-field microscopy images of guinea pig cardiomyocytes incubated for 24 h with EVs (ultracentrifuged at 100,000× *g*) from A375 melanoma cells treated with 10 nM Doxorubicin (Dox-EVs) or vehicle (Ctrl-EVs). Panel show (**a**) Ctrl-EVs at 0 min, (**b**) Ctrl-EVs at 40 min, (**c**) Dox-EVs at 0 min, and (**d**) Dox-EVs at 40 min after MTT addition. Cardiomyocytes incubated with Ctrl-EVs accumulated purple formazan over time, indicating active mitochondrial reductase activity, while Dox-EVs-treated cells showed reduced formazan formation; scale bar 100 µm. (**B**) Time-course quantification of MTT reduction over 50 min. Cardiomyocytes treated with Ctrl-EVs showed a steady increase in formazan signal, whereas Dox-EV-treated cells displayed significantly reduced kinetics. We present data as mean ± SEM (statistical) of corrected formazan intensity relative to the baseline (0 min) and over the fold change (F.C.) of Dox-EVs effect versus Ctrl-EVs effect and its *p*-value summary. (**C**) Violin plot showing MTT activity per individual cardiomyocyte after 40 min of MTT exposure. Dots represent single-cell data points; solid and dashed lines indicate median and quartiles, respectively. Cells exposed to Dox-EVs showed significantly lower MTT activity compared to Ctrl-EVs-treated cells (F.C. ≈ −2.3). We performed statistical analysis using an unpaired two-tailed Student’s *t*-test *p* < 0.01 (**), *p* < 0.0001 (****)); n = 6–9 cardiomyocytes per group. Data represent three independent experiments using cardiomyocytes from different guinea pigs and EVs isolated from separate A375 cultures (n = 3).

Bright-field microscopy (Figure 4A) revealed that cardiomyocytes incubated with control EVs (Ctrl-EVs) generated substantial intracellular formazan crystals within 40 min of MTT addition, indicating active mitochondrial metabolism. In contrast, cardiomyocytes treated with Dox-EVs displayed visibly reduced formazan accumulation, suggesting diminished metabolic activity.

We performed a time-course analysis of MTT reduction kinetics to quantify these differences (Figure 4B). Cardiomyocytes exposed to Ctrl-EVs showed a consistent and time-dependent increase in formazan production over 50 min. In comparison, those treated with Dox-EVs exhibited significantly slower formazan accumulation, with statistically significant differences emerging at 30 (F.C. ∞, *p* < 0.0001, R^2^ (η^2^) = 0.85), 40 (F.C.: = −2.25, *p* = 0.0021, R^2^ (η^2^) = 0.63), and 50 min (F.C. = −2.17, *p* = 0.0017, R^2^ (η^2^) = 0.64), indicating suppressed metabolic activity.

At the single-cell level, digital image quantification confirmed this suppression (Figure 4C). The violin plot demonstrated a significantly lower median and narrower distribution of corrected formazan intensity in Dox-EVs-exposed cardiomyocytes compared to Ctrl-EVs-exposed cardiomyocytes. These findings suggest that Doxorubicin alters the functional properties of secreted EVs, impairing their ability to sustain mitochondrial activity in cardiomyocytes. This functional shift may reflect Dox-induced changes in vesicle cargo composition and could contribute to systemic off-target effects such as cardiotoxicity.

To verify that the isolated EVs fractions were free of residual doxorubicin (Dox), we assessed Dox levels in culture media, conditioned media, and EVs fractions using UV–Vis spectrophotometry and fluorometry (excitation/emission: 480/590 nm). Standard curves were generated using serial dilutions of Dox in PBS and DMEM. The experimental Dox dose (10 nM) was below the quantification limit for UV–Vis detection and could only be detected by fluorescence.

Because the DMEM culture medium and secreted components in conditioned media generated background fluorescence and quenched the Dox signal, we applied a standard addition method to account for matrix effects. Known concentrations of Dox were introduced into the culture media, and EVs derived from Dox-exposed cells were close to zero, within the experimental error, and had values like those from control samples, suggesting the absence of detectable Dox in both conditioned media and EVs.

To assess if EVs were responsible for the change in metabolic activity of cardiomyocytes, we performed exposition experiments with EVs-enriched conditioned media and EVs-depleted conditioned media. We found that the effect observed with isolated EVs was like those obtained with conditioned media (EVs-enriched conditioned media), but the effect size was reduced to near zero in EVs-depleted conditioned media, suggesting that EVs were the responsible for observed effect.

Overall, EVs from Dox-treated A375 cells showed the capacity to change cardiomyocyte metabolism. Although Doxorubicin increased EVs secretion, the vesicles exhibited altered functional properties, likely reflecting chemotherapy-induced changes in their molecular cargo.

### 2.5. TGF-β Is Enriched in the Cargo of EVs Derived from Doxorubicin-Treated A375 Cells

**Figure 5:**
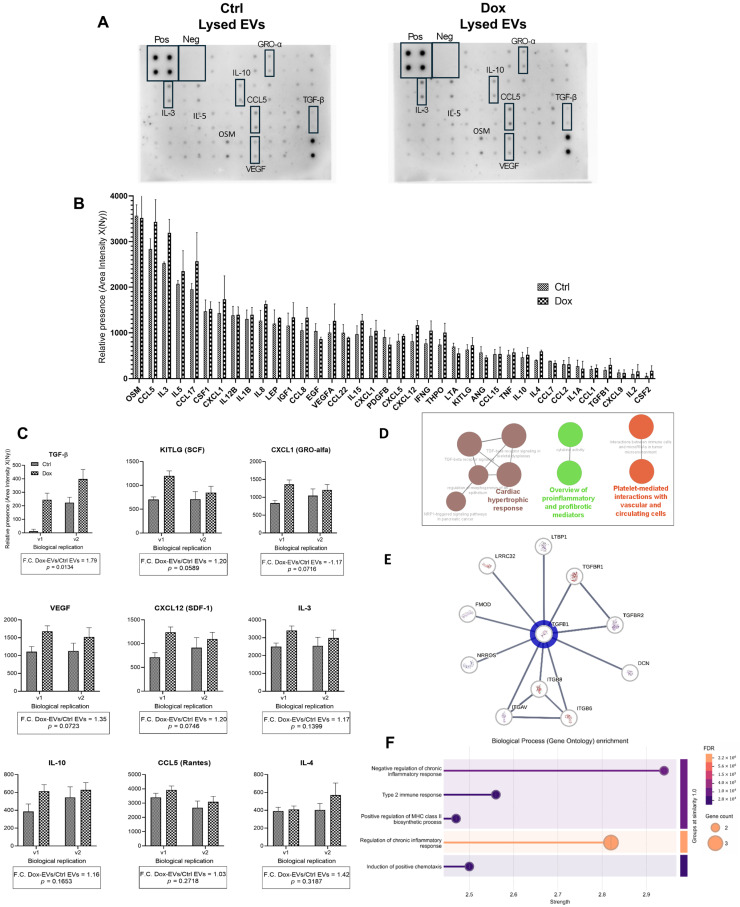
**Cytokine profiling of small extracellular vesicles derived from Doxorubicin-treated A375 melanoma cells: Functional implications of TGF-β enrichment and cytokine modulation**. (**A**) Membrane-based cytokine array. Representative images of cytokine antibody arrays used to analyze lysed EVs from control and Dox-treated A375 cells. We indicated positive and negative controls and highlighted differentially expressed cytokines with black boxes. (**B**) Global cytokine expression profile. Relative expression levels of 39/42 detected cytokines were quantified by densitometry, applying parabolic background correction (radius = 5), subtraction of background (mean of negative controls), and normalization to the mean signal of positive controls from control arrays. We present results as mean ± SEM (statistical) from two biological replicates, each with two technical replicates (n = 2). (**C**) We performed a focused analysis of key cytokines, examining the expression of TGF-β, KITLG, CXCL1, VEGF, CXCL12, IL-3, IL-10, CCL5, and IL-4 using the same normalization strategy. We applied a two-way ANOVA with Sidak’s correction to compare Dox- versus control-treated conditions for each cytokine in each replicate. We report mean values ± SEM (statistical) and indicate statistical significance accordingly. (**D**) Enriched biological processes identified through ClueGO network analysis of cytokine-related signatures associated with EVs derived from melanoma cells exposed to Doxorubicin include pathways implicated in the cardiac hypertrophic response, pro-inflammatory and profibrotic mediator activity, and platelet-mediated interactions with vascular and circulating cells. The subnetwork displayed corresponds to a focused segment of the comprehensive ClueGO network, provided in Supplementary Figure S3. (**E**) Protein–protein interaction (PPI) network constructed using STRING (2024), centered on TGFB1 (dark blue node), reveals direct associations with canonical receptors (TGFBR1, TGFBR2), latent binding partners (LTBP1), extracellular matrix components (DCN, FMOD), integrin subunits (ITGAV, ITGB6, ITGB8), and regulatory proteins (NRROS, LRRC32). The network comprises nine nodes and 30 edges, with an average node degree of 6.67 and a statistically significant PPI enrichment (*p*-value = 5.66 × 10^−15^). We supported edges with evidence from text mining, experimental data, curated databases, co-expression, genomic neighborhood, gene fusion events, and co-occurrence. We based interaction confidence on a medium threshold score (≥0.4). We used edge thickness and color intensity to denote the strength of functional association. (**F**) Gene Ontology (GO) enrichment analysis (Biological Process category) performed via STRING, with terms clustered based on semantic similarity (threshold = 1.0) and ranked by enrichment strength. We annotated each term with the corresponding false discovery rate (FDR) and the number of associated genes. Results indicate a significant upregulation of TGF-β-related processes in response to Doxorubicin, suggesting that EVs, particularly exosomes released post-treatment, may function as key mediators of tumor–stromal communication within the microenvironment.

Small extracellular vesicles released by A375 melanoma cells following treatment with 10 nM Doxorubicin exhibited a markedly different cytokine profile compared to control EVs, as shown in Figure 5. Membrane-based cytokine arrays (Figure 5A) revealed visibly stronger signals for several cytokines in the Dox-EVs group, highlighted by black boxes. The arrays included internal positive and negative controls, confirming technical reliability.

Quantitative densitometry analysis of 39 out of 42 detectable cytokines (Figure 5B) indicated a global upregulation in cytokine content in Dox-EVs. We corrected the data for background using the mean of negative controls and normalized it to the positive controls from the control array. We observed the most prominent increase for TGF-β, with a fold change (F.C.) of 1.79 ± 0.14 (*p* = 0.0134), corresponding to a large effect size (Cohen’s *d* = 1.26). KITLG (F.C. = 1.20 ± 0.21, *p* = 0.0589, *d* = 0.91), CXCL1 (F.C. = −1.17 ± 0.13, *p* = 0.0716, *d* = 0.89), and VEGF (F.C. = 1.35 ± 0.17, *p* = 0.0723, *d* = 0.84) also showed significant increases, all with large effect sizes. These values indicate robust treatment-induced cytokine enrichment.

Additional cytokines, including CCL5 (F.C. = 1.03 ± 0.15), IL-3 (F.C. = 1.17 ± 0.11), and IL-10 (F.C. = 1.16 ± 0.13), showed moderate increases with medium effect sizes (*d* = 0.78, 0.49, and 0.44, respectively); IL-4 (F.C. = 1.42 ± 0.10, *p* = 0.3187).

All data were derived from two biological replicates with two technical replicates each (n = 2) and analyzed by two-way ANOVA followed by Sidak’s correction. The overall treatment effect across cytokines yielded a large effect size (η^2^ = 0.152), supporting a substantial impact of Doxorubicin on EVs cytokine composition (Figure 5C).

Functional enrichment analysis using ClueGO (Figure 5D) revealed that the upregulated cytokines were significantly associated with biological processes such as “regulation of immune response,” “angiogenesis,” “response to wounding,” and “extracellular matrix organization,” with *p*-adjusted values < 0.001. These pathways are critically involved in tumor progression, microenvironment remodeling, and immunomodulation.

STRING-based protein-protein interaction (PPI) network analysis (Figure 5E) placed TGF-β1 at the center of a highly connected cluster of nine nodes and thirty edges, with an average node degree of 6.67 and a PPI enrichment *p*-value of 5.66 × 10^−15^. We drew interaction evidence from experimental data, curated databases, and text mining, and represented confidence levels by edge thickness.

Finally, Gene Ontology (GO) enrichment using STRING (Figure 5F) confirmed significant overrepresentation of TGF-β-associated biological processes. Key enriched terms included “positive regulation of cell migration,” “extracellular matrix organization,” and “regulation of immune effector process,” with high enrichment strength and FDR-corrected *p*-values further validating the biological relevance of the observed cytokine modulation.

Taken together, these results demonstrate that Doxorubicin significantly altered the cytokine cargo of A375-derived EVs, particularly increasing TGF-β and other key modulators of the tumor microenvironment. The stress-induced remodeling of vesicular content may contribute to pathways linked to cancer progression and have potential systemic implications, including cardiovascular effects.

## 3. Discussion

We first standardized final melanoma cell densities to eliminate variability in EVs production and composition. Under these controlled conditions, sublethal Doxorubicin (Dox) exposure induced a nonlethal stress response in melanoma cells, leading to significant alterations in EVs quantity, cargo, and function. These changes have important implications for tumor adaptation and systemic toxicity, particularly through EVs-mediated signaling involving TGF-β and other cytokines, with potential contributions to chemotherapy-induced cardiovascular toxicity.

### 3.1. Sublethal Doxorubicin Induces Controlled Cellular Stress in A375 Melanoma Cells Without Triggering Cell Death

Despite the widespread clinical use of Dox, its full spectrum of molecular effects, particularly at sublethal concentrations, remains incompletely defined. In this study, we identified 10 nM Dox as an optimal sublethal concentration that induces stress in A375 melanoma cells without triggering apoptosis or necrosis. Our data show that this dosage elicits moderate mitochondrial dysfunction without compromising membrane integrity or inducing Annexin V/PI positivity. Prior reports support these findings by highlighting low-dose Dox-induced stress responses, including senescence, oxidative stress, and metabolic reprogramming [7,28,35,36,37].

Establishing a sublethal concentration of Dox that induces cellular stress without triggering apoptotic or necrotic pathways is critical for investigating its impact on EVs biogenesis and cargo composition [35,36]. In our study, A375 melanoma cells exposed to increasing Dox concentrations exhibited a dose-dependent reduction in viability, as demonstrated by MTT assays and image cytometry. While higher concentrations (≥100 nM) significantly reduced metabolic activity and cell viability, 12.5 nM induced moderate, nonlethal effects, preserving cellular morphology and avoiding apoptotic or necrotic features. To determine the optimal sublethal dose, we tested intermediate concentrations (10–20 nM) and identified 10 nM as the lowest dose that significantly reduced metabolic activity (*p* = 0.0109), while maintaining membrane integrity and Annexin V/PI negativity—confirming the absence of cell death and validating its suitability for downstream EVs analyses.

These results are consistent with prior reports indicating that sublethal Dox concentrations can trigger cellular stress responses, including endoplasmic reticulum stress, reactive oxygen species (ROS) production, and transcriptional reprogramming, without overt cytotoxicity [38,39]. Notably, several studies have shown that this type of sublethal stress stimulates EVs biogenesis in multiple cancer models, including melanoma and breast cancer [20,40,41], potentially altering EVs cargo and modulating tumor–host interactions.

By seeding different initial densities (2.5 × 10^5^ for Dox-treated and 1.5 × 10^5^ for controls), we ensured that both groups reached comparable cell numbers (~1 × 10^6^ cells/mL) at the final 144 h time point, with <4% apoptosis/necrosis. This standardization enables accurate comparative analyses of EVs yield and quality across different conditions.

Taken together, our findings validate the use of 10 nM Dox as an optimal sublethal dose to induce controlled stress in melanoma cells, creating a reliable experimental system to explore stress-induced alterations in EVs biogenesis and secretion and downstream signaling pathways.

### 3.2. Doxorubicin Promotes Endosomal Trafficking and EVs Biogenesis in A375 Cells

Having established that 10 nM Dox induces a nonlethal but biologically active stress phenotype in melanoma cells, we next investigated whether this condition modulates vesicle trafficking pathways and stimulates EVs biogenesis.

To investigate whether low-dose Dox modulates vesicle biogenesis and intracellular trafficking pathways, we evaluated late endosomes (LEs), multi-vesicular bodies (MVBs), and Golgi-associated compartments in A375 melanoma cells. Sublethal stress induced by 10 nM Dox significantly altered vesicular structures and enhanced the formation of acidic and ceramide-rich vesicles, indicative of increased endolysosomal and exosomal activity [20,42].

Acridine orange staining revealed a notable elevation in acidic vesicular organelles 24 h post-treatment, with a significant increase in corrected total cell fluorescence (CTCF) compared to controls (F.C. = 1.72, *p* = 0.0007, R^2^ (η^2^) = 0.132), suggesting enhanced acidification and endolysosomal maturation. These findings align with studies demonstrating that stressors such as chemotherapeutic agents or oxidative insults activate the lysosomal-autophagic system and endosomal maturation as adaptive mechanisms [43,44].

At 96 h, Bodipy-TR ceramide staining revealed ceramide-enriched Golgi vesicles and increased vesicle clustering in treated cells. Ceramide plays a central role in exosome formation by promoting inward budding of MVB membranes [26]. The elevated ceramide signal (F.C. = 1.72, *p* < 0.0001) suggests Dox enhances Golgi-derived trafficking and EVs precursor generation, in line with mechanisms described in stress-induced exosome release [45,46,47].

SEM imaging (microscopy) at 24 h revealed heterogeneous vesicle populations on the surface of Dox-treated cells, with vesicle diameters ranging between 131 and 158 nm, consistent with small EVs, including exosomes [48]. This morphological evidence supports a rapid and stress-induced increase in EVs secretion, as previously observed in tumor cells exposed to sublethal chemotherapy [20,40,41].

Furthermore, immunofluorescence analysis of CD81 and Rab7 confirmed that Dox promotes activation of vesicle biogenesis pathways. CD81, a canonical exosomal marker [19,39], was significantly elevated at 96 h (*p* = 0.0083), while Rab7, involved in late endosome maturation and MVB trafficking [34], increased earlier (F.C. = 3.34, *p* = 0.348). Increased expression of CD81 and Rab7, coupled with SEM evidence of EVs release, further confirmed that Dox triggers intracellular vesicle remodeling. These findings align with studies demonstrating that chemotherapy enhances EVs biogenesis via ceramide-dependent pathways [43] under nonlethal stress. Such sublethal activation of vesicle pathways may underlie adaptive tumor responses and EVs-mediated communication during chemotherapy [12,44].

### 3.3. Isolation and Characteristics of Small Extracellular Vesicles Derived from A375 Melanoma Cells Treated with Doxorubicin

The observed increase in endolysosomal activity and EVs-associated markers strongly suggests enhanced vesicle production under sublethal chemotherapeutic stress. To validate this, we next isolated and characterized small EVs from control and Dox-treated A375 cells using a combination of high-fidelity methods aligned with the Minimal Information for Studies of Extracellular Vesicles (MISEV2023) guidelines [18].

The combination of differential ultracentrifugation and size exclusion chromatography employed in this study represents a high-fidelity strategy for isolating small extracellular vesicles with improved purity. This dual approach minimizes contamination from non-vesicular components and apoptotic bodies, which can confound downstream analyses. It also aligns with the MISEV2023 guidelines, which advocate for multiple complementary isolation and characterization techniques to ensure reproducibility and biological relevance [18,19]. The inclusion of a parallel ultracentrifugation protocol for functional studies further strengthens the rigor and comparability of the results.

Quantitative analysis using nanoparticle tracking (NTA) demonstrated a significant increase in EVs release following treatment with sublethal concentrations of Dox (10 nM). The concentration of vesicles nearly tripled in treated samples compared to controls, supported by large effect sizes (Cohen’s *d* = 106.3; R^2^ (η^2^) = 0.0052), highlighting the robustness of this finding. The increased particle concentration across SEC fractions, particularly in fractions 6 through 10, indicates a widespread activation of EVs biogenesis in response to chemotherapeutic stress. These observations are consistent with prior reports that sublethal doses of anthracyclines and other genotoxic agents enhance EVs secretion as part of a broader stress response [41,48,49], often mediated through oxidative stress and ceramide-dependent endosomal pathways [46].

Further characterization of the vesicles revealed necessary biochemical alterations. Fluorescence-based NTA using Bodipy-TR dye indicated a significant increase in ceramide-enriched vesicles in the Dox-treated group, consistent with the role of ceramide in MVB formation and exosome release [36]. Increases in the protein-to-particle ratio and the detection of Galectin-3, a marker of vesicle membrane integrity and breakdown, suggest not only an upregulation in vesicle quantity but also changes in vesicle quality and cargo content. Notably, Western blot analysis showed an increase in CD81 expression, a canonical exosomal marker, with an approximate 2.1-fold enrichment in Dox-derived vesicles. The findings further support that low doses of Doxorubicin promote the release of exosome-like vesicles through regulated biogenesis pathways and confirm qualitative changes in vesicle content and integrity [22,23].

### 3.4. Extracellular Vesicles from Doxo-Stressed Melanoma Cells Induce Metabolic Dysfunction in Cardiomyocytes

The enrichment of TGF-β and other proangiogenic cytokines in Dox-EVs prompted us to investigate their potential functional impact on nontumor cells. Given the established systemic toxicity of anthracyclines, we evaluated how these vesicles affect the metabolic integrity of primary ventricular cardiomyocytes as a model for off-target, chemotherapy-induced injury [7,40,44].

The findings presented in this section demonstrate that small extracellular vesicles released by A375 melanoma cells after sublethal Dox exposure can significantly impair the metabolic activity of primary ventricular cardiomyocytes. Using a single-cell MTT assay as a proxy for mitochondrial function, cardiomyocytes treated with EVs derived from Dox-treated melanoma cells (Dox-EVs) displayed a marked reduction in formazan formation compared to cells exposed to EVs from untreated controls. This observation, quantified via time-lapse imaging and kinetic analysis, reveals a suppression of mitochondrial reductase activity that was both time-dependent and statistically significant from 30 min onward, consistent with a dose-independent but cargo-dependent alteration of EVs bioactivity.

The observed impairment in MTT reduction suggests that Dox does not merely stimulate vesicle release but also alters the qualitative characteristics of EVs, potentially through changes in their protein, lipid, or miRNA cargo. Given that Dox is known to induce endoplasmic reticulum stress, mitochondrial dysfunction, and oxidative damage, it is plausible that this stress responses reprogram vesicle biogenesis and cargo loading mechanisms in donor tumor cells. Previous studies have demonstrated that chemotherapy-exposed tumor cells secrete EVs with distinct molecular signatures, including enhanced levels of TGF-β, heat shock proteins, and ROS-associated metabolites, which can exert deleterious effects on distant nontumor cells [35,37]. The present results are consistent with this concept, as the Dox-EVs appear to transmit stress signals to cardiomyocytes, leading to metabolic dysregulation.

Importantly, this study leverages single-cell resolution to reveal heterogeneity in cardiomyocyte responses to EVs exposure. Violin plot analysis revealed a narrower distribution and a significantly lower median formazan intensity in Dox-EVs–treated cells, indicating not only a global reduction in mitochondrial function but also a loss of functional resilience across individual cells. The fold change in MTT activity was approximately 2.3, underscoring the magnitude of the effect and its potential biological relevance. These results were consistent across three independent biological replicates, using cardiomyocytes from different animals and EVs from separately treated melanoma cultures, which reinforces the reproducibility and robustness of the findings.

From a broader perspective, these data provide a mechanistic link between tumor-derived extracellular vesicles and systemic off-target effects of chemotherapy, particularly within the cardiovascular system. Anthracyclines like Dox are well-established inducers of cardiotoxicity, with mechanisms involving mitochondrial damage, redox imbalance, and fibrosis [20,34]. However, the role of tumor-secreted EVs as mediators of this toxicity has only recently emerged as an area of investigation. The results presented here support the hypothesis that EVs act not only as carriers of tumor-promoting signals but also as vehicles of systemic toxicity, capable of disrupting cellular energetics in distant tissues such as the myocardium.

Given the central role of mitochondrial integrity in cardiomyocyte viability and contractility, the reduction in MTT reductase activity raises concerns about longer-term consequences, including contractile dysfunction, increased susceptibility to apoptotic stimuli, and eventual cardiac remodeling. These findings warrant further investigation into the specific molecular cargo of Dox-EVs, particularly redox-active metabolites, mitochondrial RNAs, or miRNAs regulating energy homeostasis (e.g., miR-34a, miR-210) and how they interact with cardiomyocyte signaling pathways [24,44]. Additionally, functional studies assessing calcium handling, mitochondrial membrane potential, and ROS production would provide further insight into the exact mechanisms by which EVs mediate their deleterious effects [7].

This study provides compelling evidence that EVs released from melanoma cells under chemotherapeutic stress exhibit altered functionality and can negatively impact the metabolic state of primary cardiomyocytes. These findings highlight a novel dimension of cancer–host communication and suggest that monitoring or modulating EVs content may hold potential in mitigating chemotherapy-associated cardiac side effects. Integrating EVs-based biomarkers or inhibitors of EVs biogenesis into cardio-oncology strategies could open new avenues for improving patient outcomes during cancer treatment.

### 3.5. TGF-β-Enriched EVs from Doxorubicin-Treated Melanoma Cells: Functional and Bioinformatic Insights into Cytokines’ Roles in Cancer Progression and Cardiovascular Impact

Beyond quantitative changes in EVs output, our findings revealed shifts in vesicle composition indicative of stress-adaptive cargo remodeling. We therefore assessed the cytokine content of these vesicles to explore whether Dox-induced EVs convey specific pro-tumorigenic or systemic cardiovascular modulators such as TGF-β.

Using membrane-based cytokine arrays, the study found a broad upregulation of cytokines in Dox-EVs compared to controls, with TGF-β levels rising nearly 1.8-fold and exhibiting a large effect size. Other cytokines such as KITLG, CXCL1, and VEGF also showed significant increases, pointing to a coordinated remodeling of vesicle cargo in response to chemotherapeutic stress.

The data presented reveal that sublethal exposure to Dox significantly alters the cytokine composition of EVs derived from A375 melanoma cells, with a particularly pronounced enrichment of TGF-β. This finding is especially relevant in the context of melanoma, where TGF-β plays a key role in promoting tumor plasticity, immune escape, and metastatic progression, while also contributing to cardiovascular complications such as fibrosis and inflammation through EVs-mediated systemic signaling under Dox-induced stress [13,30]. This cytokine shift may have substantial functional implications, as TGF-β promotes epithelial–mesenchymal transition (EMT), immune evasion, and the formation of pre-metastatic niches [26,39,40].

TGF-β enrichment in EVs suggests that Dox, while exerting cytotoxic pressure, may paradoxically prime tumor cells to modulate their microenvironment and facilitate progression through EVs-mediated paracrine signaling. VEGF and CXCL1 are potent proangiogenic factors, and their co-enrichment with TGF-β reinforces the notion that these vesicles could enhance vascular remodeling in the tumor stroma or at distant metastatic sites [20,41]. Likewise, the presence of KITLG and CXCL12 in Dox-EVs aligns with pathways involved in stem cell niche activation and tumor–stromal crosstalk.

Gene Ontology enrichment and protein–protein interaction analyses validate the cytokine data, confirming that the upregulated factors associate with biological processes such as angiogenesis, immune regulation, wound response, and extracellular matrix organization. Notably, TGF-β1 occupies a central position in a high-confidence interaction network, reinforcing its biological importance within the EV cargo. These findings align with previous reports showing that EVs act as vectors for cytokine delivery and functional modulation of recipient cells, particularly under stress or treatment conditions [7,41,50,51].

The observed changes also raise questions regarding the systemic effects of these vesicles beyond the tumor. Circulating TGF-β-enriched EVs may affect distant tissues, including the myocardium, potentially contributing to the fibrotic and inflammatory responses seen in anthracycline-induced cardiotoxicity [29,30]. In this context, the Dox-induced remodeling of EVs content could bridge cancer–host interactions with off-target side effects, expanding the functional reach of the vesicles beyond the immediate tumor microenvironment.

The integration of cytokine profiling with GO and PPI network analysis strengthens the evidence that EVs-associated cytokines [52], particularly TGF-β, are upregulated in response to sublethal Dox exposure. Taken together, these results suggest that chemotherapy not only selects resistant tumor cells but may also reprogram their secretory landscape, enabling pro-tumorigenic and potentially profibrotic signaling via extracellular vesicles. This dual impact underscores the importance of profiling EVs cargo as part of the systemic response to cancer therapy. It highlights the potential of TGF-β-bearing vesicles as biomarkers or therapeutic targets in both oncology and cardio-oncology.

These molecular and quantitative changes in vesicle production are of particular interest given their potential biological implications. Chemotherapy-altered EVs may play dual roles, both locally within the tumor microenvironment and systemically in distal organs. In cancer, stress-enhanced EVs secretion has been associated with increased tumor aggressiveness, through the delivery of oncogenic proteins, TGF-β, and immunomodulatory miRNAs that promote stromal remodeling and immune evasion [35].

In melanoma specifically, vesicles may act on fibroblasts, endothelial cells, and immune populations to reinforce a tumor-supportive niche. Systemically, these vesicles could also contribute to adverse effects of chemotherapy, such as cardiotoxicity, through the delivery of profibrotic or pro-inflammatory signals, including TGF-β, to cardiac tissues. This is especially relevant given the established cardiotoxic risk profile of anthracyclines like Dox, which may exert indirect effects via circulating EVs [29,30].

Taken together, these findings provide evidence that sublethal Dox exposure not only enhances the biogenesis of extracellular vesicles in melanoma cells but also modifies their biochemical composition in a manner consistent with stress-adaptive responses. The study supports the growing view that EVs are not passive byproducts but active mediators of intercellular communication during chemotherapy.

Further investigation into specific cargo, particularly TGF-β isoforms, miRNAs, and fibrosis-related proteins, is warranted to elucidate their role in promoting cancer progression or distant tissue damage, such as in the heart. This study highlights TGF-β as a central node in networks related to fibrosis, immune modulation, and vascular remodeling. Profiling these vesicles and testing their functional effects on recipient cells such as cardiomyocytes, endothelial, or fibroblasts cells will be essential for understanding their contribution to the systemic side effects of chemotherapy and their potential as therapeutic targets or biomarkers.

To contextualize the observed biological effects of Dox-derived EVs, we conducted a bioinformatic analysis of vesicle cargo and associated signaling pathways. This approach enabled the identification of central molecular mediators, particularly TGF-β, and their roles in bridging tumor stress responses with systemic outcomes such as fibrosis, immune modulation, and cardiotoxicity.

Comprehensive bioinformatic analyses identified Fibromodulin (FMOD) and NRROS as relevant mediators potentially driving EVs-associated systemic effects. FMOD, a proteoglycan of the extracellular matrix, modulates collagen fibrillogenesis and influences TGF-β bioavailability, linking it to cardiac fibrosis [30]. NRROS, a negative regulator of reactive oxygen species, suppresses TGF-β signaling and inflammatory activation, thereby acting as a counter-regulatory element during tissue remodeling and stress [29].

STRING-based PPI networks centered on TGF-β revealed high-confidence connectivity with fibrotic, angiogenic, and immunomodulatory cytokines such as VEGF, Galectin-3, and CXCL1, which the Dox-treated melanoma cells also enriched in their released EVs [30,40].

The pleiotropic nature of TGF-β is particularly significant. While TGF-β may exert tumor-suppressive effects during early melanoma development [36], under sublethal chemotherapy stress, it assumes a more pro-metastatic and fibrogenic phenotype, facilitating epithelial–mesenchymal transition and fibrotic tissue remodeling [20,38].

Our analysis of SEC fractionation profiles and CD81+ EVs populations indicates that Doxorubicin exposure not only increases EVs output but also alters vesicle composition and subtype, suggesting functional heterogeneity among released vesicles. These findings are in line with reports describing chemotherapy-induced EVs remodeling [15,37].

Our cardiomyocyte assays demonstrate that Dox-EVs impair mitochondrial metabolism, a hallmark of early anthracycline-induced cardiotoxicity [7,24,42]. Because TGF-β and Galectin-3 both contribute to cardiac fibrosis and remodeling [20,37], their presence in EVs cargo supports a plausible mechanism for vesicle-mediated myocardial injury.

These findings converge with broader models of doxorubicin-induced cardiomyopathy, in which ROS overproduction, NF-κB and MAPK activation, and TGF-β1-driven myofibroblast differentiation contribute to interstitial collagen deposition, ventricular stiffening, and eventual heart failure [49]. In addition, EVs-mediated delivery of TGF-β and related cytokines may amplify these processes, especially within the context of vascular calcification and atherosclerotic plaque maturation [30].

Our findings indicate that TGF-β-enriched EVs function as context-dependent mediators, bridging tumor biology with systemic cardiovascular toxicity. These results reinforce the concept that chemo-EVs are dynamic entities bearing distinct molecular signatures, which may serve as potential biomarkers of treatment response [35].

Additionally, sublethal exposure of melanoma cells to Dox triggers an adaptive vesicle-mediated response that influences both the tumor environment and systemic functions. Additionally, sublethal exposure of melanoma cells to Dox triggers an adaptive, vesicle-mediated response that reshapes both the tumor environment and systemic functions. Under these conditions, the cells release EVs enriched in profibrotic and immunomodulatory cytokines—particularly TGF-β—that alter cardiac cell metabolism and may contribute to cardiovascular dysfunction.

### 3.6. Limitations

This study provides new insights into how sublethal Dox exposure reprograms melanoma cells to enhance extracellular vesicles biogenesis and modify their molecular cargo. Notably, these vesicles accumulate TGF-β and other cytokines that influence tumor progression and affect nontumoral tissues such as cardiomyocytes. Although validation by ELISA or Western blot for selected cytokines (TGFβ, VEGF, CXCL1, KITLG) could not be performed due to current reagent limitations, the reproducibility observed across independent biological and technical replicates strengthens confidence in these preliminary findings. This limitation also provides a clear direction for future studies aimed at confirming and expanding the cytokine signatures identified here.

Our data show that these stress-induced EVs alter mitochondrial metabolism in primary cardiomyocytes, highlighting their potential role in systemic chemotherapy-associated toxicity. The integration of functional assays and bioinformatic analyses positions these vesicles as active participants in both local tumor adaptation and distant organ dysfunction. While these results offer novel insights, several limitations must be acknowledged and warrant further consideration.

First, we conducted all experiments in a single melanoma cell line (A375), which limits the ability to capture the full genetic and phenotypic diversity of melanoma. We deliberately chose A375 because, like other melanoma models, it produces high levels of EVs, which do not capture the full genetic and phenotypic diversity of melanoma.

We deliberately chose A375, as this melanoma model—like others—exhibits high extracellular vesicle shedding activity [38], making it a suitable platform to investigate vesicle remodeling. In previous work, we demonstrated that sublethal doxorubicin exposure induces distinct EVs and cytokine profiles in a non-melanoma model (MCF7 breast cancer) [7].

Here, we show that A375 cells also change EVs cargo, albeit with a different cytokine signature. While these findings suggest that stress-induced EVs modulation may occur across cancer types, our conclusions are specific to A375. Future studies should validate these observations in genetically distinct melanoma lines (e.g., BRAF wild-type) and patient-derived xenografts, as well as in non-melanoma models, making it a suitable platform to investigate vesicle remodeling.

Second, we performed functional assays using primary ventricular cardiomyocytes in ex vivo models, and there is currently no in vivo evidence confirming that Dox-induced EVs reach cardiac tissue or mediate organ-level toxicity. Animal studies using tumor-bearing models and in vivo EV tracking techniques (e.g., PKH-labeled vesicles) would be essential to validate systemic biodistribution and target organ interactions.

Third, mitochondrial metabolic activity was the sole functional readout in cardiomyocytes. Although the MTT assay provides a proxy for reductase activity, it does not inform about mitochondrial membrane potential, ROS production, or calcium flux. We recommend employing a broader panel of functional assays, such as JC-1 staining, Seahorse XF bioenergetic profiling, or fluorescent Ca^2+^ indicators, to better delineate mitochondrial dysfunction and apoptosis in cardiomyocytes exposed to chemotherapeutic agents [7]. These methods more precisely characterize mitochondrial impairment. These approaches could uncover additional mechanistic insights into EVs-induced cardiotoxicity.

Fourth, although we applied rigorous EVs purification protocols—including sequential washing steps after Dox exposure, ultracentrifugation, and size exclusion chromatography, we cannot entirely exclude the possibility that trace amounts of residual Dox or soluble contaminants may co-isolate with EVs.

In our study, we did not intentionally package Dox into EVs; instead, we evaluated the biological effects of EVs released by cells exposed to a sublethal concentration of Dox (10 nM). We included extensive washing steps (Section 2.1) to minimize the risk of Dox contamination. Nonetheless, future work should consist of additional control experiments using Dox-treated media without cells or media spiked with free Dox to confirm that the observed effects depend on EVs.

Finally, we have not thoroughly explored the potential heterogeneity of vesicle subpopulations. Distinguishing between exosomes, microvesicles, and other EVs subtypes may provide further resolution in identifying which specific vesicle class contributes to observed biological effects. Future studies should consider implementing techniques such as immunocapture using subtype-specific markers (e.g., CD63, CD81, Annexin A1), density gradient centrifugation, or high-resolution flow cytometry to isolate and analyze distinct EVs populations. This approach may uncover subtype-specific roles in mediating tumor and cardiovascular effects.

Additionally, the unique cytokine and protein profile of Dox-EVs, characterized by elevated levels of TGF-β, Galectin-3, and profibrotic mediators such as IL-11 and OPN, suggests the potential use of circulating vesicles as dynamic biomarkers for chemotherapy-induced toxicity. These markers are not only implicated in cardiac fibrosis and remodeling but are detectable in patients in patients’ plasma, making them also detectable. Longitudinal patient studies comparing EVs cargo with clinical markers of cardiac injury (e.g., NT-proBNP, troponin) may reveal correlations functional for early detection and patient monitoring. We based our findings on an in vitro cardiomyocyte model exposed to EVs derived from melanoma cells; therefore, we urge caution when extrapolating these results to the complex in vivo context of anthracycline-induced cardiotoxicity, where tissue microenvironment, systemic metabolism, and immune modulation may significantly alter EV composition and biological effects.

### 3.7. Future Directions

Building on these findings, we will investigate the precise molecular mediators within Doxorubicin-induced EVs that drive their systemic effects. We plan to characterize the miRNA, lncRNA, and protein cargo of Dox-EVs with cardiotoxic potential using small RNA sequencing and EVs proteomics. Investigation of redox-active metabolites and fibrosis-related molecules may also help explain the vesicle-mediated impact on cardiac physiology. Together, these molecular components may drive the mitochondrial dysfunction and reduce the metabolic activity observed in treated cardiomyocytes.

Expanding the study to include additional melanoma cell lines with diverse mutational backgrounds or primary melanoma cultures could improve translational relevance. Moreover, the use of in vivo tumor-bearing models will be crucial for assessing the distribution, uptake, and physiological impact of Dox-EVs within the context of an intact cardiovascular system.

Testing pharmacological inhibitors of EVs biogenesis (e.g., GW4869, targeting neutral sphingomyelinase) or TGF-β antagonists in vitro and in vivo may help define strategies to mitigate off-target toxicities. Functional rescue experiments using cardiomyocytes pretreated with such inhibitors could validate the contribution of EVs cargo to the observed metabolic dysfunction.

Researchers should also explore sex-based and age-related variability in cardiotoxic susceptibility, both in vitro using cardiomyocytes from donors of different demographics and in vivo using representative animal models.

Future research should compare the biological effects and recipient cell responses to EVs derived from Dox-exposed cells versus EVs actively loaded with Dox, to clarify the functional distinctions and therapeutic implications of each approach. Although this study did not address it, higher, clinically relevant concentrations of chemotherapeutics may lead cells to passively or actively package Dox into EVs.

A growing body of literature shows that EVs can serve as natural nanocarriers for drugs like Dox, offering enhanced stability, reduced systemic toxicity, and targeted delivery. We acknowledge the potential of EVs-mediated drug delivery systems for both oncologic and cardio-oncologic research. Future studies should determine whether EVs from cells treated with higher Dox concentrations encapsulate the drug, and assess how this affects their biodistribution, therapeutic efficacy, and off-target effects [7,49,50].

Taken together, these future directions offer a pathway to deepen mechanistic understanding and support the development of cardio-oncology tools that integrate EVs biology with precision medicine approaches.

### 3.8. Potential Clinical Applications

Despite these limitations, the data presented here open new avenues for translational research at the intersection of oncology and cardiovascular medicine. By uncovering how chemotherapy-induced stress reshapes the EV landscape to include bioactive cargo such as TGF-β, our study demonstrates the dual roles of EVs as mediators of tumor adaptation and systemic toxicity [29,35]. These insights provide a conceptual foundation for exploring EVs as both biomarkers of chemotherapeutic response and potential therapeutic targets aimed at mitigating off-target organ damage.

Our findings may open new avenues for novel EVs-targeted strategies in cardio-oncology. Inhibiting EVs biogenesis (e.g., via ceramide pathway blockade) [26] or neutralizing specific cargo (e.g., TGF-β antagonists) could mitigate chemotherapy-induced organ damage. Furthermore, profiling circulating EVs may aid in the early detection of cardiotoxicity, supporting the development of precision monitoring tools for patients undergoing anthracycline-based therapy [12,43,44]. Their dynamic profiles, shaped by chemotherapeutic stress, underscore their dual roles as biomarkers of therapeutic stress and potential therapeutic targets themselves. Future research should characterize the miRNA, lncRNA, and protein cargo of Dox-EVs with cardiotoxic potential. Furthermore, animal models and clinical cohorts are crucial for translating these findings into patient care [48]. Investigating their effects on endothelial and immune cells could clarify roles in atherogenesis.

## 4. Materials and Methods

### 4.1. Cell Culture and Doxorubicin Treatment

We cultured human melanoma A375 cells (American Type Culture Collection, Manassas, VA, USA; ATCC^®^ CRL-1619) in high-glucose Dulbecco’s Modified Eagle Medium (DMEM; Gibco™, Cat. No. 11966025, Thermo Fisher Scientific, Waltham, MA, USA) supplemented with 10% heat-inactivated fetal bovine serum (FBS; Gibco™, Cat. No. 16140, Thermo Fisher Scientific, Grand Island, NY, USA) and 1% penicillin–streptomycin (Gibco™, Cat. No. 15140122). We maintained the cells at 37 °C in a humidified incubator with 5% CO_2_ and passaged them at 70–80% confluency, following standard melanoma culture procedures [40].

For experimental treatments, we exposed cells to sublethal concentrations of doxorubicin hydrochloride (10–100 nM; Sigma-Aldrich, Cat. No. D1515; manufactured in Bengaluru, Karnataka, India) for 96 h. We selected this dosing regimen based on previous studies showing metabolic stress without apoptosis induction [5,27]. We diluted Dox in culture medium and used 0.1% (*v*/*v*) dimethyl sulfoxide (DMSO; MP Biomedicals™, Cat. No. 0219058280, Solon, OH, USA) as the vehicle control.

After treating the A375 cells with Dox, we washed the cells twice and collected the conditioned medium after 48 h. We isolated small EVs using sequential centrifugation, ultrafiltration, and size exclusion chromatography or ultracentrifugation. We examined the morphology of the EVs using scanning electron microscopy (SEM), determined their size and particle concentration through Nanoparticle Tracking Analysis (NTA), determined protein concentrations with Bicinchoninic Acid (BCA) Protein Assay Kits, and evaluated some EVs markers through dot and Western blotting.

### 4.2. Cell Viability, Apoptosis, and Necrosis

#### 4.2.1. MTT Assay

We seeded A375 cells (3 × 10^3^ cells/well) in 48-well plates and treated them for 96 h, following standard viability protocols [49]. We then incubated the cells with MTT reagent (0.83 mg/mL in PBS; Sigma-Aldrich, Cat. No. M2128) for 1 h at 37 °C. After removing the supernatant, we added 100 µL of DMSO (Merck, Cat. No. 1.02952.1000, Darmstadt, Germany) to solubilize the formazan crystals [50]. We measured absorbance at 570 nm (reference 750 nm) using a GloMax^®^ Multi+ Detection System (Promega, Madison, WI, USA) and normalized the values to vehicle-treated controls.

#### 4.2.2. Apoptosis and Necrosis by Image Cytometry

Apoptosis and necrosis were assessed using the Tali Apoptosis Kit, based on Annexin V/PI staining (Annexin V Alexa Fluor 488 and propidium iodide; Thermo Fisher Scientific, Cat. No. A10788) according to the manufacturer’s protocol. We analyzed stained cells using the Tali™ Image-Based Cytometer (Thermo Fisher Scientific, Waltham, MA, USA) and calibrated fluorescence thresholds with unstained and single-stained controls [51].

### 4.3. Microscopy

#### 4.3.1. Live Cell Imaging

We stained the cells with acridine orange (Sigma-Aldrich, Cat. No. A6014) or BODIPY™ TR ceramide (Thermo Fisher Scientific™, Cat. No. D7540) to visualize acidic vesicles and multi-vesicular bodies (MVBs). Researchers have previously used ceramide staining to monitor stress-induced exosome formation in real time [36]. We performed confocal microscopy with a ZEISS LSM 900 equipped with Airyscan and a Plan-Apochromat 10×/0.45 objective (Carl Zeiss AG, Oberkochen, Germany). We processed the images using Zeiss ZEN Blue software, version 3.4 [44]

In vivo levels of vesicle markers were quantified by acridine orange and Bodipy-TR staining, followed by corrected total cell fluorescence (CTCF) calculation: CTCF = Integrated Density − (Area of selected region × Mean fluorescence of background). This quantitative analysis encompassed 40–50 cells at each time point.

#### 4.3.2. Immunocytochemistry

We used primary antibodies against canonical EV markers (CD9, CD63, CD81, TSG101) and the endosomal protein Rab7. We fixed cells in 4% paraformaldehyde (Electron Microscopy Sciences, Hatfield, PA, USA) and permeabilized them with 0.1% Triton X-100. After blocking with 3% BSA, we incubated the cells overnight at 4 °C with primary antibodies against CD9-FITC and CD63-PE (ImmunoTools GmbH, Friesoythe, Germany), Rab7 (Santa Cruz Biotechnology, Cat. No. sc-376362, Dallas, TX, USA.), CD81 (Abcam, Cat. No. ab79559, Cambridge, UK), and TSG101 (Abcam, Cat. No. ab83, Cambridge, UK). We then applied the corresponding Alexa Fluor 488 or 594 secondary antibodies (Thermo Fisher Scientific, Cat. Nos. A28175 and A21235) and counterstained the nuclei with DAPI (Sigma-Aldrich) [45]. We mounted coverslips in Gelvatol medium and performed confocal microscopy using a ZEISS LSM 900 with Airyscan (Carl Zeiss AG, Oberkochen, Germany) equipped with a Plan-Apochromat 63×/1.4 Oil DIC M27 objective. We acquired all images using 3.5% laser power and a gain of 750, and processed them with Zeiss ZEN Blue software, version 3.4 [53,54].

We quantified EV marker expression using corrected total cell fluorescence (CTCF), calculated as CTCF = Integrated Density − (Area of selected region × Mean fluorescence of background). We measured EV markers at each time point using ImageJ 1.53t, visualized the data in violin plots, and represented each point as an individual cell. We performed statistical comparisons using a two-tailed unpaired Mann–Whitney test (n = 6), indicating statistical significance as follows: * *p* < 0.05, ** *p* < 0.01, *** *p* < 0.001, ** *p* < 0.0001.

### 4.4. Isolation and Characterization of Small Extracellular Vesicles

#### 4.4.1. EVs Isolation and Purification

We switched the cells to serum-free medium to avoid FBS-derived EV contamination. We collected conditioned media (CM) and sequentially centrifuged it at 300× *g* for 10 min and 4500× *g* for 20 min at 4 °C to remove cells and debris. We filtered the supernatants through 0.22 μm Steriflip GP filters (MilliporeSigma, Burlington, MA, USA) and concentrated those using Amicon Ultra-15 filters (10 kDa cutoff; MilliporeSigma). We then isolated EVs either by ultracentrifugation at 100,000× *g* for 90 min using a Sorvall™ WX Ultra Series 90 centrifuge with a TH-641 rotor (Thermo Fisher Scientific™) or by size exclusion chromatography (SEC) with qEV10 columns (Izon Science Ltd., Oxford, UK), following MISEV2023 recommendations [18,55].

#### 4.4.2. Scanning Electron Microscopy (SEM)

We fixed the isolated EVs in 4% paraformaldehyde, dehydrated them with graded ethanol, and sputter-coated them with gold using a Quorum Q150R ES system (Quorum Technologies Ltd., Laughton, UK), following morphological standards for EV studies [35,46]. We captured images with an FEI Quanta 200 scanning electron microscope (Thermo Fisher Scientific™, Hillsboro, OR, USA) [54].

#### 4.4.3. Nanoparticle Tracking Analysis (NTA)

We measured particle size and concentration using a NanoSight NS300 system (Malvern Instruments Ltd., Worcestershire, UK) equipped with a 532 nm laser and an sCMOS camera. For each sample, we acquired five 60 s videos. We analyzed the data with NTA Software v3.4.4, using consistent settings for camera level (13 FPS) and detection threshold (4 BU) [43,54].

#### 4.4.4. Cytokine Profiling

We determined the protein concentration of EV fractions using the Pierce™ BCA Protein Assay Kit (Thermo Fisher Scientific™, Cat. No. 23227). We profiled cytokine content with the Human Cytokine Antibody Array C5 (Abcam, Cat. No. ab133997), which detects 42 cytokines—ENA-78, GCSF, GM-CSF, GRO, GRO-alpha, I-309, IL-1alpha, IL-1beta, IL-2, IL-3, IL-4, IL-5, IL-6, IL-7, IL-8, IL-10, IL-12 p40/p70, IL-13, IL-15, IFN-gamma, MCP-1, MCP-2, MCP-3, MCSF, MDC, MIG, MIP-1delta, RANTES, SCF, SDF-1, TARC, TGF-beta1, TNF-alpha, TNF-beta, EGF, IGF-I, Angiogenin, Oncostatin M, Thrombopoietin, VEGF-A, PDGF BB, and Leptin—following a method previously used to characterize EV-associated inflammatory signatures [7]. We detected the signals via chemiluminescence and imaged them with the ChemiDoc MP System (Bio-Rad, Hercules, CA, USA). We quantified the signals using Fiji/ImageJ v1.53q. A detailed workflow appears in the Supplementary Materials

### 4.5. Cardiomyocyte Isolation and Functional Assays

We isolated ventricular cardiomyocytes from adult guinea pigs (*Cavia porcellus*, 250–300 g) as previously described [7,50,56], following institutional ethical guidelines. After inducing deep anesthesia, we excised the hearts and perfused them retrogradely with Ca^2+^-free M199 medium (Sigma-Aldrich) containing EGTA, then performed enzymatic digestion with pronase E (Sigma-Aldrich), proteinase K (Promega), and collagenase type II (Sigma-Aldrich).

We resuspended rod-shaped viable cardiomyocytes in Tyrode’s solution and exposed them to purified EVs (0.025–2.5 µg/mL protein) or conditioned media for 24 h at 30 °C. We assessed cell morphology, MTT reduction, and formazan crystal formation using a Nikon Eclipse Ti-S inverted microscope (Nikon Corporation, Tokyo, Japan). We analyzed the images with NIS-Elements D.3.22.00 and Fiji/ImageJ software, evaluating a minimum of six cardiomyocytes per field.

### 4.6. Bioinformatic Analysis

We analyzed protein–protein interaction networks and pathway enrichment using STRING v10 (DTU, Lyngby, Denmark) and visualized the results in Cytoscape v3.9.1 with the ClueGO v2.5.10 and CluePedia v1.5.10 plug-ins. We performed enrichment analyses based on the Gene Ontology Biological Process (GO-BP) and WikiPathways databases, following approaches used in EV cargo enrichment studies [57,58,59]. We considered Bonferroni-adjusted *p*-values < 0.05 statistically significant.

### 4.7. Statistical Analysis

We presented the data as mean ± standard error of the mean (SEM) and performed statistical analyses using GraphPad Prism v8.0 (GraphPad Software Inc., San Diego, CA, USA). We assessed normality of distribution with the Shapiro–Wilk test. For two-group comparisons, we applied either Student’s *t*-test (parametric) or the Mann–Whitney U test (non-parametric). For multiple group comparisons, we used one-way or two-way ANOVA followed by Holm–Šidák’s post hoc test. We considered results statistically significant when *p* < 0.05. We estimated effect sizes using Cohen’s *d* for *t*-tests, eta-squared (η^2^) for ANOVA, and regression model R^2^ (η^2^) for unpaired *t*-tests with unequal group sizes, as in the cardiomyocyte assays. We interpreted effect sizes as small (Cohen’s *d* = 0.2–0.49; η^2^ = 0.01–0.059; R^2^ = 0.2–0.49), medium (Cohen’s *d* = 0.5–0.79; η^2^ = 0.06–0.139; R^2^ = 0.5–0.79), or large (Cohen’s *d* ≥ 0.8; η^2^ ≥ 0.14; R^2^ ≥ 0.8) according to established statistical frameworks [7].

## 5. Conclusions

Our study describes that sublethal exposure to Doxorubicin reprograms the extracellular vesicles profile of melanoma cells, enhancing their biogenesis and enriching their cargo with profibrotic and immunomodulatory mediators, notably TGF-β. These altered vesicles exert effects beyond the tumor microenvironment, impairing mitochondrial metabolism in cardiomyocytes and potentially contributing to vascular inflammation, metabolic dysregulation, and cardiac fibrosis. By integrating cellular, molecular, and computational approaches, we highlight the dual role of EVs in promoting tumor adaptation and mediating off-target toxicity. These findings underscore the relevance of EVs as both mechanistic drivers and potential biomarkers of chemotherapy-induced systemic alterations and point to vesicle-targeted strategies as promising avenues to mitigate adverse effects while improving therapeutic outcomes in cancer and cardiovascular contexts.

## Data Availability

We include all supporting information as Supplemental Material, attaching Supplementary Figures and NanoSight Tracking Analysis (NTA) videos to this article.

## Supplementary Materials

The following supporting information can be downloaded at https://www.mdpi.com/article/10.3390/ijms26178524/s1.
